# Comparative effect of intraoperative propacetamol versus placebo on morphine consumption after elective reduction mammoplasty under remifentanil-based anesthesia: a randomized control trial [ISRCTN71723173]

**DOI:** 10.1186/1471-2253-4-6

**Published:** 2004-09-14

**Authors:** Michèle Binhas, François Decailliot, Saïda Rezaiguia-Delclaux, Powen Suen, Marc Dumerat, Véronique François, Xavier Combes, Philippe Duvaldestin

**Affiliations:** 1 Service d'Anesthésie-Réanimation, Hôpital Henri Mondor, AP-HP, Université Paris XII, 51, avenue du Maréchal de Lattre de Tassigny, 94010 Créteil, France

## Abstract

**Background:**

Postoperative administration of paracetamol or its prodrug propacetamol has been shown to decrease pain with a morphine sparing effect. However, the effect of propacetamol administered intra-operatively on post-operative pain and early postoperative morphine consumption has not been clearly evaluated. In order to evaluate the effectiveness of analgesic protocols in the management of post-operative pain, a standardized anesthesia protocol without long-acting opioids is crucial. Thus, for ethical reasons, the surgical procedure under general anesthesia with remifentanil as the only intraoperative analgesic must be associated with a moderate predictable postoperative pain.

**Methods:**

We were interested in determining the postoperative effect of propacetamol administered intraoperatively after intraoperative remifentanil. Thirty-six adult women undergoing mammoplasty with remifentanil-based anesthesia were randomly assigned to receive propacetamol 2 g or placebo one hour before the end of surgery. After remifentanil interruption and tracheal extubation in recovery room, pain was assessed and intravenous titrated morphine was given. The primary end-point was the cumulative dose of morphine administered in the recovery room. The secondary end-points were the pain score after tracheal extubation and one hour after, the delay for obtaining a Simplified Numerical Pain Scale (SNPS) less than 4, and the incidence of morphine side effects in the recovery room.

For intergroup comparisons, categorical variables were compared using the chi-squared test and continuous variables were compared using the Student t test or Mann-Whitney U test, as appropriate. A *p *value less than 0.05 was considered as significant.

**Results:**

In recovery room, morphine consumption was lower in the propacetamol group than in the placebo group (p = 0.01). Pain scores were similar in both groups after tracheal extubation and lower in the propacetamol group (p = 0.003) one hour after tracheal extubation. The time to reach a SNPS < 4 was significantly shorter in the propacetamol group (p = 0.02). The incidence of morphine related side effects did not differ between the two groups.

**Conclusions:**

Intraoperative propacetamol administration with remifentanil based-anesthesia improved significantly early postoperative pain by sparing morphine and shortening the delay to achieve pain relief.

## Background

Postoperative administration of paracetamol or its prodrug propacetamol has been shown to decrease pain with a morphine sparing effect [1-4]. The effect of propacetamol administered intraoperatively on postoperative pain and early postoperative morphine consumption has not been clearly evaluated. However, for a predictable moderate postoperative pain, intraoperative administration of non-opioid analgesics such as paracetamol and postoperative intravenous administration of morphine are recommended in patients undergoing general anesthesia with remifentanil [5]. Indeed, remifentanil differs from potent mu agonists by its extremely short elimination half-life [6]. The elimination kinetics of remifentanil is so fast that its analgesic effect wears off abruptly, thus making the management of postoperative pain critical.

In order to evaluate the effectiveness of intraoperative paracetamol administration in the management of postoperative pain and morphine consumption, a standardized anesthesia protocol without long-acting opioid is necessary. Thus, for ethical reasons, the surgical procedure under general anesthesia with remifentanil as the only intraoperative analgesic must be associated with a moderate predictable postoperative pain.

Therefore, the present study was designed to evaluate the effect of intraoperative administration of propacetamol during remifentanil-based anesthesia on postoperative pain in patients undergoing reduction mammoplasty.

## Methods

### Patients

After approval by the Local Ethical Committee and written informed consent, 36 consecutive female patients who underwent elective reduction mammoplasty were included. Exclusion criteria were the preoperative use of analgesic drugs; a body mass index ≥ 35, an American Society of Anesthesiology physical status ≥ 3 and sensitivity to paracetamol. Pain evaluation using a Simplified Numerical Pain Scale (SNPS) was explained to the patients during the preoperative anesthetic visit and the day before surgery. A standardized surgical technique was used for all patients.

### Anesthetic protocol

Hydroxyzine 2 mg.kg^-1 ^was given orally 12 h and 2 h before anesthesia as premedication. Before induction of anesthesia, patients were randomly allocated to either Placebo group or Propacetamol group. Randomization was based on computer-generated codes maintained in sequentially numbered, opaque envelopes. The preparation of the propacetamol or saline infusion was done by a nurse who was not in charge of the patient. The anesthetist, the patient and the nurse's staff caring for the patients in the recovery room were unaware of the treatment group. In both groups, anesthesia was induced with propofol 2.5 mg.kg^-1 ^followed by a slow bolus (1 min) of remifentanil 1 mcg.kg^-1^. Tracheal intubation was facilitated by atracurium 0.5 mg.kg^-1^. Anesthesia was maintained with remifentanil 0.1 mcg.kg^-1^.min^-1 ^and isoflurane (0.5–1.0% end-tidal) with nitrous oxide (N_2_O) in 50% oxygen. Remifentanil infusion rate was increased or decreased by 0.05 mcg.kg.min^-1 ^in order to maintain an arterial systolic pressure of 20% more or less than the baseline value. One hour before the end of surgery, which corresponded to the beginning of the skin closure, patients received either propacetamol 2 g in 50 cc saline (Propacetamol group) or 50 cc saline alone (Placebo group) infused over 10 min. At the end of surgery, administration of isoflurane and N_2_O were withdrawn and the patient was transferred in the recovery room. The anaesthetist, the patient and the nurse's staff caring for the patients in the recovery room were unaware of the treatment group. Remifentanil infusion was interrupted when the patient arrived in recovery room. Tracheal extubation was performed within a few minutes after remifentanil discontinuation. Clinical monitoring included heart rate, blood pressure, pulse oxymetry, respiratory rate, and sedation score (0: awake, 1: drowsy and 2: asleep).

From the time of extubation, pain was evaluated on using a SNPS (from 0, no pain to 10 the worse pain) and intravenous 2 mg morphine was administered on request every 5 min until pain relief (SNPS<4). When pain relief was reached, SNPS was subsequently evaluated every 15 min. Morphine was interrupted when the sedation score went up to 1, systemic arterial pressure < 80 mmHg or respiratory rate less than 8/min. During the data collection period, intravenous morphine titration was further administered if SNPS was up to 4. Patients did not receive antiemetic prophylaxis. If post-operative nausea and vomiting (PONV) occurred, metoclopramide 10 mg and ondansetron 4 mg if necessary were intravenously administered.

Patients fulfilling Aldrete criteria [7] were discharged from recovery room.

### Measurements

Morphine requirement, pain and sedation scores were measured every 5 min until pain relief was obtained. When SNPS was below 4 during 15 min, parameters were subsequently recorded every 15 min. Morphine side effects (nausea, vomiting, urinary retention, shivering and itching) and need for supplemental medications (e.g., antiemetics) were also recorded.

The total dose of morphine in recovery room was the primary end point. Pain scores after extubation and one hour after tracheal extubation, delay for morphine requirement, delay for pain relief and incidence of morphine side effects were recorded.

### Statistical analysis

Data are expressed as mean (± standard deviation) for quantitative variables normally distributed, or otherwise as median (25th – 75th percentiles) when data were not normally distributed, and as percentage for categorical variables. Data were analyzed using Statview 5.0 software (SAS Institute Inc, USA). For intergroup comparisons, categorical variables were compared using the chi-squared test and continuous variables were compared using the Student t test or Mann-Whitney U test, as appropriate. A *p *value less than 0.05 was considered as significant. Anticipating a standard deviation of 2.49 [8], it was calculated that 15 patients at least were necessary to show a difference between groups in morphine consumption of 4 mg (considered as a clinically relevant difference) with a 80% power and a 5% type 1 error.

## Results

Thirty-six patients were included during a 12 months period: 19 in Propacetamol group and 17 in Placebo group (Table 1). There was no significant difference between the groups concerning clinical characteristics, anesthesia duration and total amount of remifentanil administered. In all patients, extubation was obtained within 7 ± 3 min (Table 1) after remifentanil discontinuation.

**Table 1:** Demographic characteristics and perioperative parameters.

	**Placebo Group (*n *= 17)**	**Propacetamol Group (*n *= 19)**
Age (years)	41(16)	34 (14)
Body mass index (kg.m^-2^)	26 (4)	25 (3)
Duration of anesthesia (min)	245 (55)	245 (80)
Remifentanil consumption (μg.kg^-1^.min^-1^)	0.150 (0.055)	0.155 (0.040)
Final intraoperative corporeal temperature (Celsius)	36.8 (0.4)	36.7 (0.5)
Delay before extubation after remifentanil interruption (min)	7 (3)	7 (3)

Data are expressed as mean (standard deviation) for age, body mass index, final intraoperative corporeal temperature and delay before extubation. Other data are expressed as median (interquartile).

In recovery room, cumulative morphine consumption was significantly lower in the Propacetamol group than in the Placebo group (Table 2). Five minutes after extubation, pain scores were similar in both groups (SNPS = 6). Pain scores one hour after tracheal extubation were significantly lower in the Propacetamol group than in the Placebo group (Table 2). Moreover, the time to reach a SNPS score less than 4 was significantly shorter in Propacetamol group compared to the Placebo group. All the patients received intravenous morphine titration in the first hour after extubation and three patients (one in Placebo group and two in Propacetamol group) required a morphine titration over one hour after extubation. Once a SNPS value less than 4 was obtained, pain scores remained stable and similar in both groups except in one patient in Placebo group who required an additional 2 mg intravenous bolus of morphine 80 min after extubation.

**Table 2:** Postoperative intravenous morphine requirement and pain scores in recovery room.

	**Placebo Group (*n *= 17)**	**Propacetamol Group (*n *= 19)**	***p *value**
Morphine consumption in recovery room (mg)	16 [8–34]	10 [6–28]	0.01
Delay between extubation and first morphine administration (min)	5 [5–20]	5 [5–15]	NS
SNPS score five minutes after extubation	6 [0–9]	6 [0–10]	NS
SNPS score one hour after extubation	3 [2–6]	2 [0–4]	0.003
Delay between extubation and obtaining a SNPS score < 4 (min)	40 [20–85]	30 [15–70]	0.02

Data are expressed as median [25^th^–75^th ^percentile]. P < 0.05 was considered as statistically significant. Pain score was evaluated on using a Simplified Numerical Pain Scale (SNPS: from 0, no pain to 10 the worse pain).

The incidence of morphine adverse effects was similar in both groups: 5 patients had nausea (3 in Propacetamol and 2 in Placebo group, p=NS) and 4 patients had vomiting (2 in each group, p=NS), no other side effects were observed. In one patient receiving placebo, morphine titration was interrupted because of nausea and vomiting.

## Discussion

This study shows that propacetamol administered one hour before end of surgery reduced the morphine dose given over the first four postoperative hours and shortened the elapsed time to obtain a SNPS under 4 in patients undergoing elective reduction mammoplasty. In our study, surgical technique and anesthetic protocol were similar in both groups. Remifentanil was the only analgesic used during anesthesia period and was interrupted before morphine administration. Thus, the beneficial effect on postoperative pain observed is clearly linked to intraoperative administration of propacetamol.

In a recent study [9], Verchère and colleagues failed to demonstrate a postoperative analgesic effect of intraoperative propacetamol administration, after remifentanil anesthesia for supratentorial craniotomy. Pain after supratentorial neurosurgery was too severe and paracetamol was insufficient to relief it. In our study mammoplasty was chosen because postoperative pain is moderate [10], and intravenous administration of morphine was used in recovery room. Thus, the morphine sparing effect of intraoperative administration of paracetamol could be really evaluated with respect of ethical requirement.

Our results are apparently at variance with those of other previous studies. Paracetamol given rectally immediately after induction of anesthesia [11] or at the end of gynecological surgery [12] and orally before surgery [13-16] failed to improve early postoperative analgesia. The negative results of these studies may be ascribable to several causes such as the low initial pain score in the control group [11,12,15,17], and the difference in the route used for paracetamol administration [11-16]. An other hypothesis to consider is the use of long-acting opioid such as fentanyl that might have contributed to the early post-operative analgesia [14,18].

Cobby and colleagues observed a morphine sparing effect when paracetamol 1.3 g was administered rectally at the end of hysterectomy [19]. One explanation is that plasma concentration after 1.3 g paracetamol was sufficient to achieve opioid sparing effect comparable with that of intravenous propacetamol. This hypothesis is strengthened by the results of another trial showing that after rectal administration of a high dose of paracetamol (40 and 60.mg kg^-1^) pain score and analgesic demand were significantly reduced in the early postoperative period [20].

The presently observed incidence of postoperative nausea and vomiting was of 25% and was similar to that of a previous study [21]. Despite the reduced dose of postoperative morphine in the Propacetamol group, the incidence of postoperative nausea and vomiting was not diminished. Our results are in agreement with the recent study of Aubrun and co-workers [4] who observed that postoperative intravenous propacetamol allowed a morphine-sparing effect but did not reduce the incidence of morphine-related adverse effects in patients undergoing general surgery.

Propacetamol was administered at the beginning of skin closure, which corresponds to one hour before the end of surgery. This delay may be insufficient to achieve pain control immediately after tracheal extubation, as the peak effect of intravenous propacetamol was shown to occur only two hours after its administration [22]. Nevertheless, we considered that it was not feasible to administer propacetamol earlier, as in our practice duration of surgery was unpredictable.

In summary, intraoperative propacetamol administration in women undergoing reduction mammoplasty improved significantly early postoperative pain in recovery room, and should be recommended for postoperative pain management.

## Pre-publication history

The pre-publication history for this paper can be accessed here:


